# Situating the Embodied Mind in a Landscape of Standing Affordances for Living Without Chairs: Materializing a Philosophical Worldview

**DOI:** 10.1007/s40279-016-0520-2

**Published:** 2016-03-17

**Authors:** Erik Rietveld

**Affiliations:** Department of Philosophy/ILLC/Academic Medical Center/Amsterdam Brain and Cognition, University of Amsterdam, Oude Turfmarkt 141, 1012 GC Amsterdam, The Netherlands

## Abstract

Sitting too much is unhealthy, but a widespread habit in many societies. Realizing behavioral change in this area is hard. Our societies promote being seated via the way its places are structured: they are filled with chairs for example. How can we make healthier environments that invite people to move around more? This article shows how philosophical research in the area of embodied/enactive cognitive science let to a built vision for the office of the future, of 2025. Multidisciplinary studio RAAAF [Rietveld Architecture-Art-Affordances] and visual artist Barbara Visser built this world without chairs, titled The End of Sitting. This large rock-like landscape integrates many affordances for standing. Affordances are the possibilities for action provided by the environment. This landscape of standing affordances allows people to work standing while being supported by the material structure of the environment. This unorthodox working landscape is both an enactive art installation and the materialization of a philosophical worldview that understands people as embodied minds situated in a landscape of affordances. It stimulates reflection on the way built environments can naturally invite more active and healthy behavior.

## Key Points

**Table:** 

In our many societies almost the entirety of our surroundings have been designed for sitting, while evidence from medical research suggests that too much sitting has adverse health effects.
The philosophy of embodied cognitive science suggests that the possibilities for action provided by the material environment structure our behavior. People can generate behavioral change by radically changing these environmental affordances in the places they spend their lives.
The architectural art installation The End of Sitting presents a thinking model for living without chairs: a landscape of possibilities for supported standing that increases bodily activity and well being.

## Introduction

Office workers are addicted to sitting. We sit even though we read every day in the newspapers that “sitting kills” or that “sitting is the new smoking”. We like the comfort of chairs and, in countries like The Netherlands, the United States and Australia at least, are living in a sitting society. We sit at the breakfast table, we sit in the car, we sit in the cinema, and we sit in front of our laptop computers. One scientific study on the sitting epidemic [7] followed over 220,000 Australians to investigate the relationship between sitting time and all cause mortality. It found that those who sit 11 h or more per day have a 40 % higher risk of dying in the next 3 years than those who sit 4 h or less. Even when one exercises every day, one does not compensate for the many hours spent seated (for a review and meta-analysis of studies on the health effects of sitting see Biswas et al. [8]). Why is sitting unhealthy? Van der Ploeg et al. [7, p. 497] argue that at least one major reason is that it reduces metabolic function because of the lack of movement involved.

Why do people typically sit down when they enter a place, say an office? Why do they sit, even though many people already know that sitting too much is unhealthy? People sit because the places in which they spend their lives are *structured around being seated*. In fact, in a European country like The Netherlands, for example, the entire society is structured around sitting: offices, movie theaters, cars, schools and restaurants are filled with chairs. In public transport, in a train, for example, one feels *unlucky* if one cannot sit. In our society we even use standing as a punishment for children, we make them stand in the corner.

Let us assume that sitting is as unhealthy as the above-mentioned studies [7, 8] claim. How then can architects make an environment that invites people to alternate physical postures and break the inactivity of sitting? The objective of this article is to present an alternative for sitting, taking the perspective of the philosophy of embodied/enactive cognitive science [1, 2, 5, 9], and show how these philosophical ideas have actually materialized in a new environment. The term ‘enactive’ refers to the non-cognitivist paradigm within the philosophy of cognitive science that takes insights from the phenomenological tradition seriously (e.g., Merleau-Ponty’s work [17]) and suggests that it is skilled engagements with the environment in concrete situations that should be the starting point for understanding the cognition of living organisms [2, 5, 6, 9, 18]. An enactive art installation like The End of Sitting aims to place visitors temporarily in a world that is different from the one they normally take for granted. In this case affordances for sitting have been replaced by the (initially at least) disorienting landscape of affordances for supported standing in which the person is embedded and that engage different abilities than those normally used.

## The Philosophy of Affordances

One of the main findings of our own philosophical research on embodied cognition in everyday life and expertise is that it is not explicit thoughts or explicit intentions that drive our skilled actions but relevant *affordances* [3, 10, 11]. Affordances are the possibilities for action offered to us by the environment [1, 4–6, 12]. The floor affords walking, a cup affords grasping and a chair affords sitting. However, a chair also affords moving and leaning on. So a particular aspect of the environment can offer a multiplicity of possibilities for action.

In recent philosophical work ([6], p. 335) we have argued for a more precise definition of affordances as relations between (a) aspects of the socio-material environment, and (b) abilities available in a ‘form of life’ [13] (for more traditional accounts of affordances, see Withagen and Caljouw [14] or Chemero [1]). The notion of a ‘form of life’ comes from the work of Wittgenstein [13] and refers to a kind of animal (say lions, earthworms or humans) as characterized by the regular patterns in its behavior; to its regular ways of doing things [6]. In our human case, the form of life includes a variety of socio-cultural practices. This definition of affordances suggests that it should be possible to *piggyback* on peoples’ existing abilities for standing, leaning and hanging to create new affordances for working in all sorts of supported postures. This philosophical insight was, as it were, the basis for the creation of the architectural art installation. It motivated the architects to investigate different ways in which people in their daily lives (say at stations, in coffee bars, airports, etc.) work standing in postures scaffolded by the material environment.

We distinguish affordances available in a form of life or ecological niche from *relevant affordances* or ‘solicitations’ for a particular individual in a concrete situation [10]. Solicitations are relevant affordances or invitations for action [15, 16]. When an individual encounters an affordance that matters to him or her, for example because using it costs almost no energy, it can generate a state of bodily action readiness [11]. This solicitation-related bodily readiness is why chairs can ‘suck us in’. If we radically change the affordances available in a certain place, we will be able to generate behavioral change. Architects and artists are able to realize such a change in the built environment by creating new affordances.

## Exploring Affordances for Supported Standing

What would our world look like if we did away with chairs and *standing* became the new norm? We, that is, multidisciplinary studio RAAAF [Rietveld Architecture-Art-Affordances] and visual artist Barbara Visser, have started experimenting with affordances that support standing in different ways, including supported leaning and hanging. RAAAF was founded in 2006 by architect Ronald Rietveld and philosopher Erik Rietveld, the author of this article. Barbara Visser has a long standing interest in work at the intersection of visual art, architecture and science and is Chair of the Society of the Arts, founded by the Royal Netherlands Academy of Arts and Sciences (KNAW).

The first space we have tried to re-imagine is the office of the future. The starting point for this project was an invitation by the Chief Government Architect of The Netherlands and the Dutch Ministry of Internal Affairs to develop a vision for the office of 2025. We were surprised to find out that current plans for the workplace of the future completely ignored the mounting evidence on detrimental health effects of sedentary behavior: all of the Ministry’s plans for the future took desks and chairs as the starting point. RAAAF has developed the design methodology of strategic interventions. “Strategic interventions are precisely chosen and carefully designed interventions in city or landscape that set a ‘desired development’ in motion.” (Rietveld et al. (2014), p. 80; see chapter 3 of that book [22] for more on RAAAF’s design method). In this case we wanted to make people aware of the discrepancy between the architectural practice of making spaces that take sitting for granted and the growing evidence that sitting too much is unhealthy [7, 8]; and, moreover, to use an affordance-based architectural design approach to develop an architectural art installation that shows how people could live without chairs in the future.

**Fig. 1:**
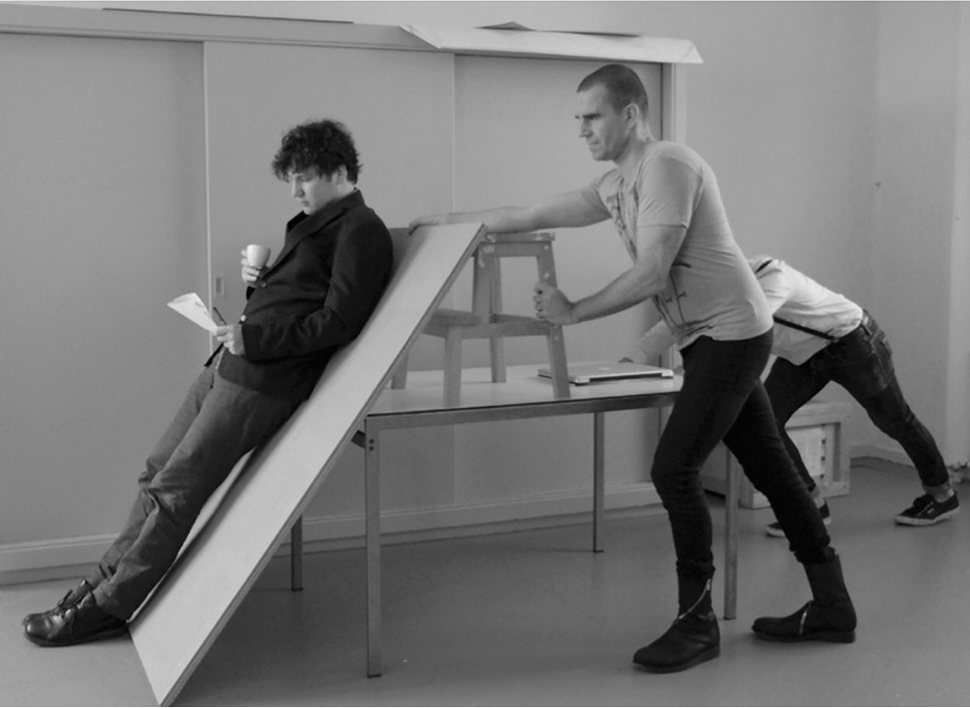
Experimentation to find an optimal angle for support of the upper body while standing. This is similar to the tendency towards an optimal grip on available affordances discussed in Merleau-Ponty [17], Dreyfus [16] and Bruineberg and Rietveld [11]. This tendency is a primarily phenomenological notion that refers to the way skilled individuals tend to adjust their postures—and activities, more broadly—to the way their surroundings are structured. In this case, the person improves his relationship to the aspect of the working environment in which he is situated by telling the people who regulate the steepness of the plank that supports his upper body which angles feel better, worse and optimal for reading while standing. Photo reproduced with permission from Barbara Visser

**Fig. 2:**
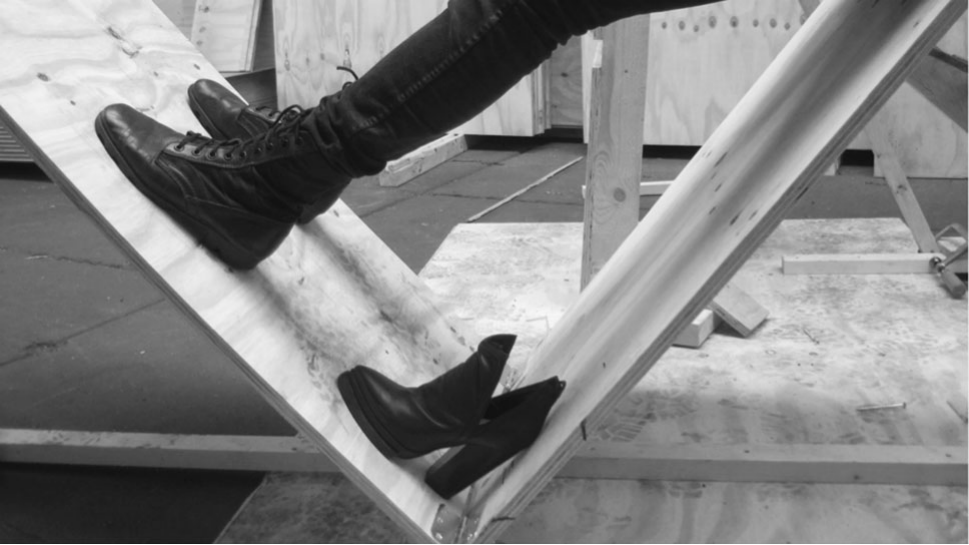
Experimentation to discover unconventional affordances for supported standing. Slanted support for feet is experienced as comfortable when combined with a scaffolded leaning position. When seeking a right angle for the planks that support parts of the body (e.g., feet and upper body), people’s experiences of better and worse angles typically have an affective component. For example, when support for their feet is too flat they will often experience dissatisfaction whereas foot supports that form 90° angles with the planks that support their upper body typically give an optimal grip, feel much better and reduce this discontent (see also the discussion of discontent experienced by architects presented in an earlier work [10]). Photograph reproduced with permission from Rietveld Architecture-Art-Affordances

Figures 1 and 2 show some of the architectural experiments the author conducted together with the RAAAF project team and visual artist Barbara Visser in order to find out what feels good in a world without chairs. The aim of these playful investigations was to discover unconventional affordances that can support us while standing at work.

**Fig. 3:**
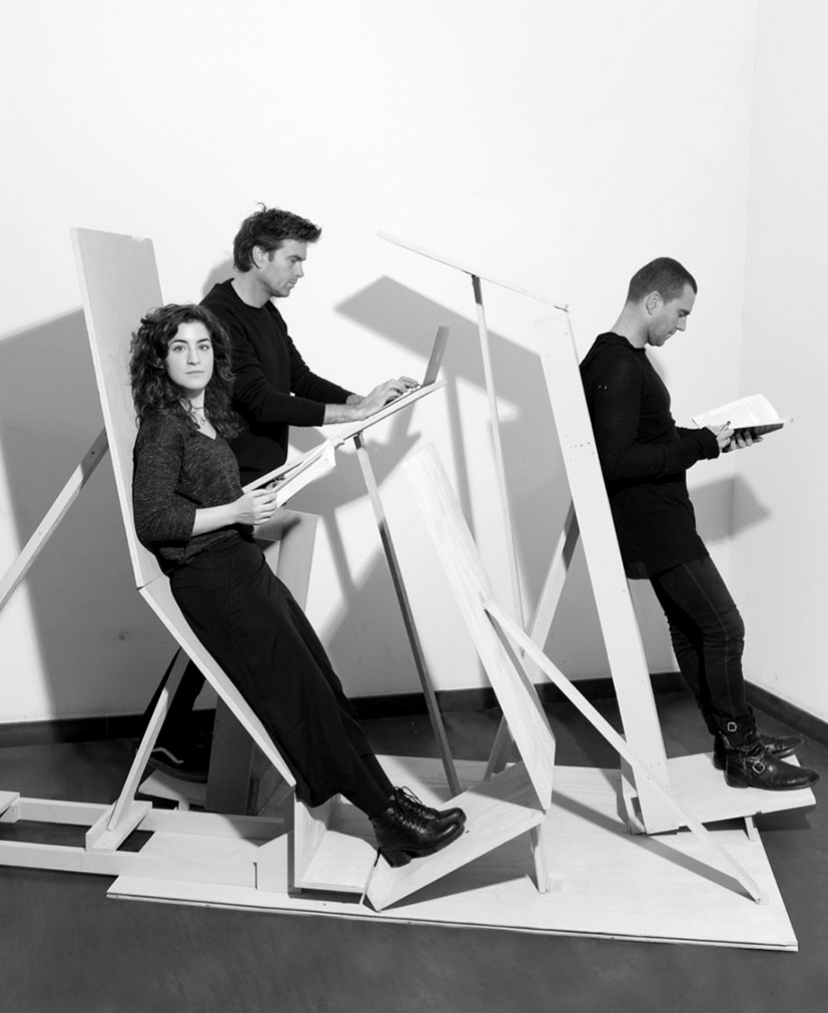
The first built prototypes of positions for the End of Sitting. Photograph reproduced with permission from Maarten Kools

While chairs have been improved on thousands and thousands of times, supported standing has long been neglected and is still open to exploration. Our philosophy of affordance and, more in particular, the resulting re-definition of affordances [6] suggests different ways in which one can discover new affordances for supported standing. One can manipulate or transform material aspects of the environment, finding out what that material can do [19]. In such a process of experimentation we can detect unexpected affordances for supported standing and leaning (Figs. 1, 2, 3). Another way to enrich the landscape of affordances, which we will investigate in future research, is by introducing new abilities in the form of life. An example could be a transfer of skills from the practice of sky diving to that of office working. One way to realize this is by looking in an entirely different form of life for unorthodox abilities that could be used to enrich the landscape of affordances (this kind of importation of an ability from a traditionally different domain is similar to what Sennett [20] calls a ‘domain shift’).

**Fig. 4:**
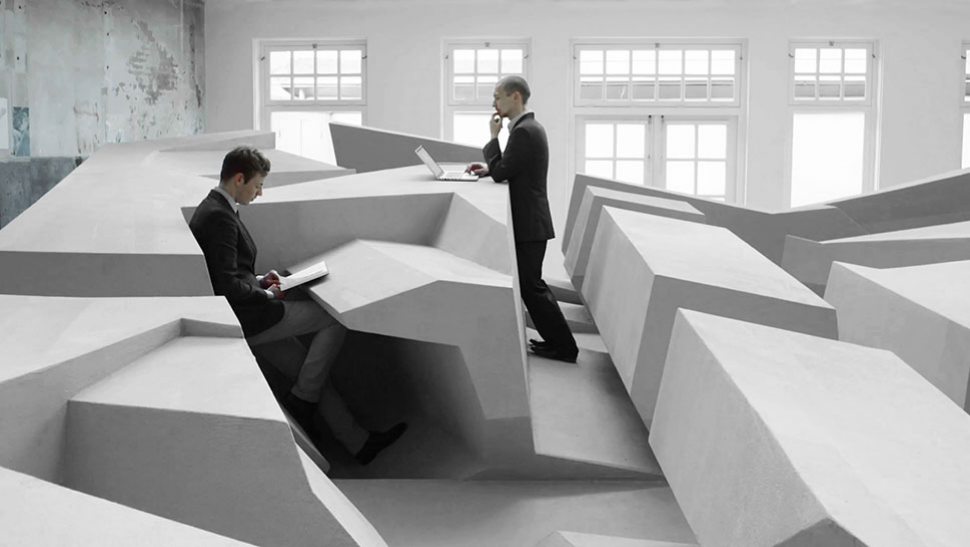
Use of two different positions in the End of Sitting landscape. Still from the film the end of sitting 1:1, reproduced with permission from Barbara Visser and Benito Strangio (camera). This film can be viewed at: https://vimeo.com/123212930

The first prototypes we built using the results of our architectural experiments can be seen in Fig. 3. The best positions we discovered came together in this art installation provocatively titled The End of Sitting (Fig. 4). This is a large experimental landscape of standing affordances.

**Fig. 5:**
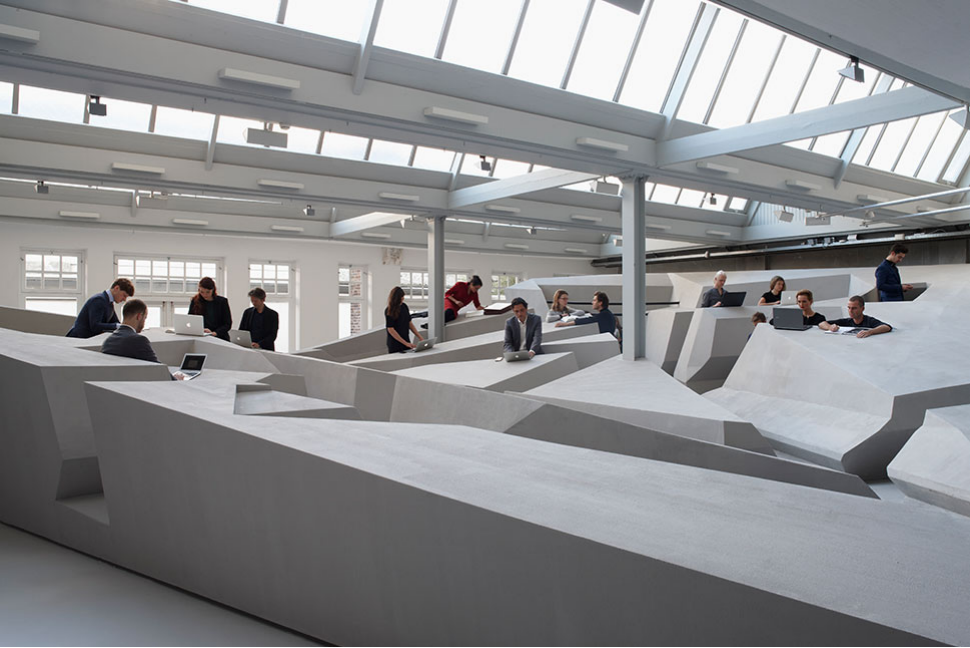
The End of Sitting. Photograph reproduced with permission from Jan Kempenaers

**Fig. 6:**
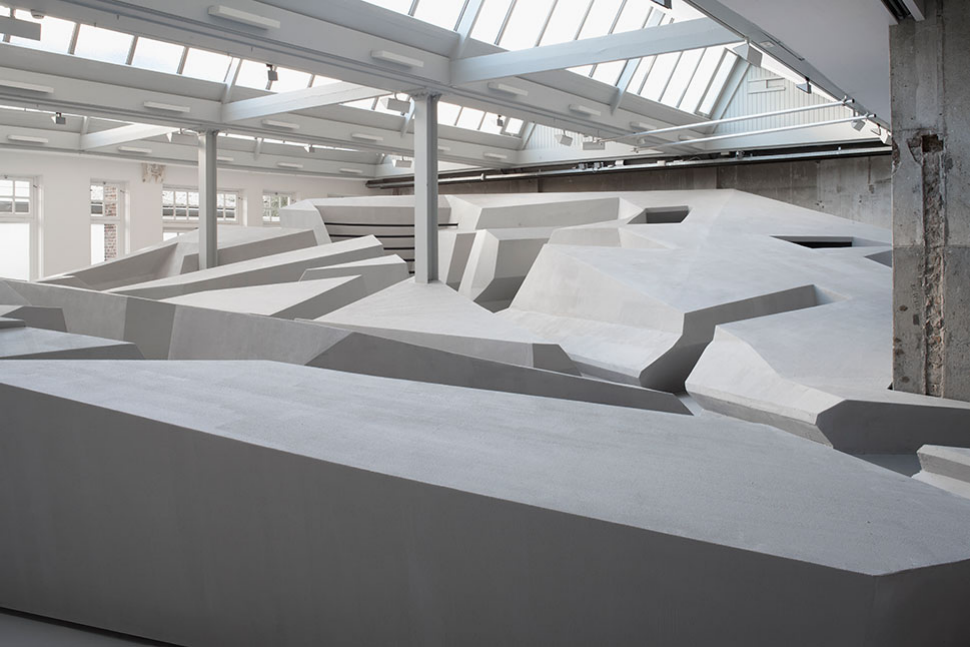
The End of Sitting sculpture. Photograph reproduced with permission from Jan Kempenaers

**Fig. 7:**
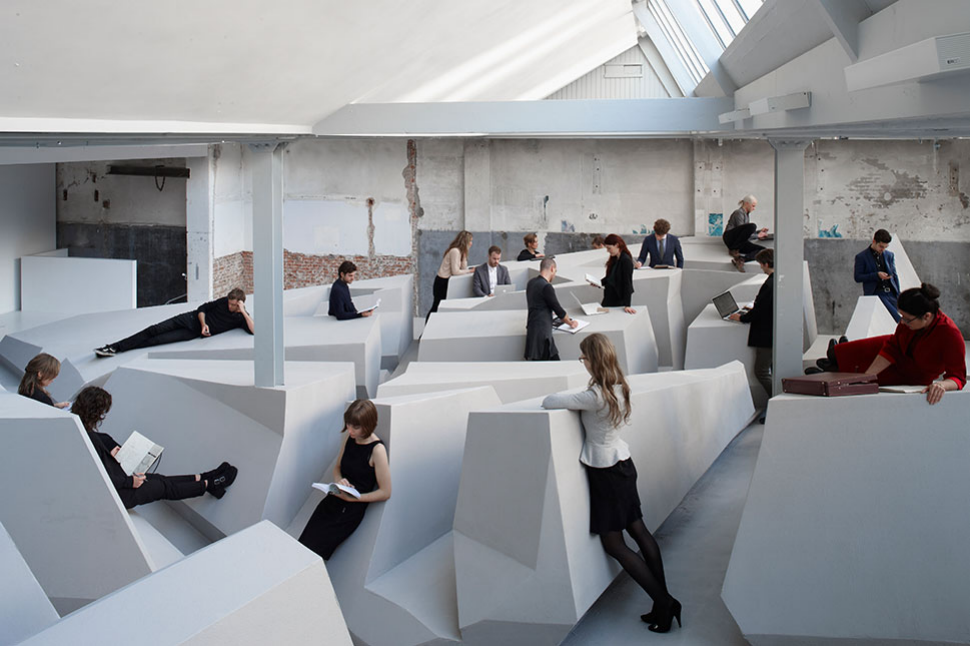
The End of Sitting. Photograph reproduced with permission from Jan Kempenaers

Figure 4 shows the use of two different positions for working while standing. The one on the right is similar to a conventional standing desk, but the one on the left is much ‘smarter’. Unlike a traditional standing desk it offers support for one’s back and provides tilted support for one’s feet. It is comfortable but not too comfortable. Each position offers temporary comfort. The End of Sitting does not offer positions that afford working comfortably in a quasi-motionless way for hours and hours, like office chairs typically do. While standing in the position on the left in Fig. 4, the largest muscle group of the body—in the legs—is constantly active. If one is seated, one’s large leg muscles are not being used, whereas while standing in The End of Sitting one’s legs will get tired after about 30 min or an hour, and the person will switch to one of the many other positions in the landscape that fits better with the current body state. Perhaps she will be lying down for a short spell, or hanging with her arms over the horizontal black ‘ropes’ that support the upper body (see Figs. 4, 5, 6, 7).

It is this dynamic of alternation of position that means that one will not stay in the same posture all day, which would be unhealthy as well. To facilitate and invite this alternation we aimed to build an entire *landscape* of affordances with many different attractive positions.

From reflection by ecological psychologist Rob Withagen and colleagues (Jongeneel et al. [23]) on an earlier RAAAF project that aimed to invite children to move by providing alluring affordances for climbing, we had learned that one way to generate locomotion was by offering a large variety of affordances. The advantage of this variety in affordances offered is that people with different abilities and body sizes would be optimally supported by the material structure. In The End of Sitting, this variety of affordances was realized by making a landscape that gradually increases in size. For many of the positions, both tall and short people would be able to easily find several soliciting spots somewhere on the rock of standing affordances.

Figures 5 and 6 show the entire sculpture as it was built in November 2014. This will not be the final version of The End of Sitting landscape. This is just the start of a long experimental trial phase up to 2025. We will continue experimenting and make it more inclusive for elderly, blind people and people with other disabilities over time.

## Living Without Chairs

What is it like to live without chairs in The End of Sitting installation? Empirical research by Rob Withagen and Simone Caljouw of the University of Groningen investigates how people use and experience this landscape. Some of the research questions in that study were: Do the subjects become more energetic? What does working in the landscape mean for their wellbeing?

**Fig. 8:**
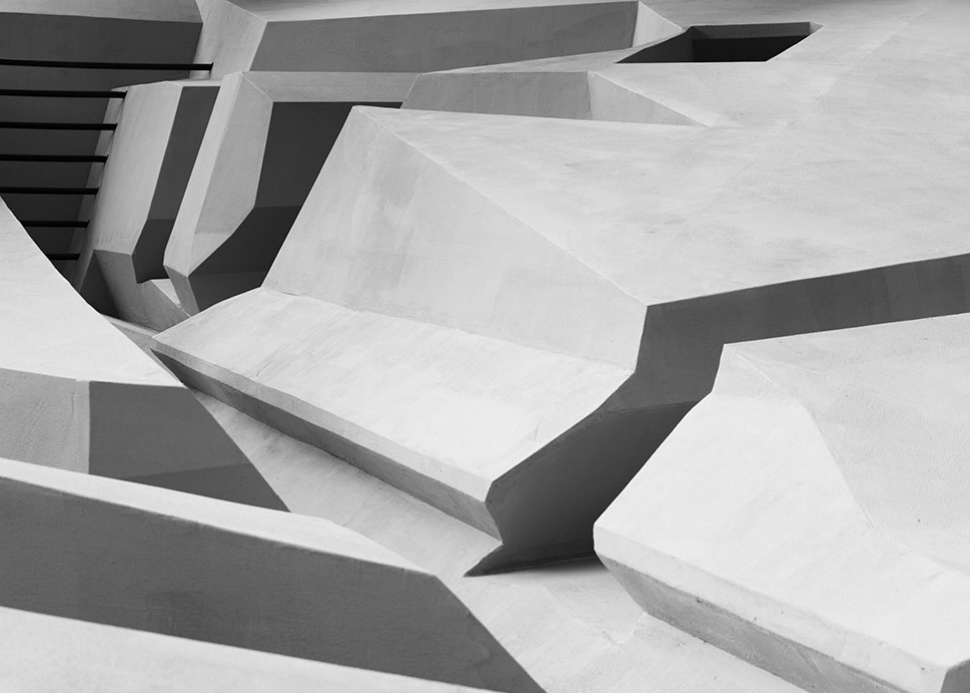
The End of Sitting—a closer view. Photograph reproduced with permission from Ricky Rijkenberg

**Fig. 9:**
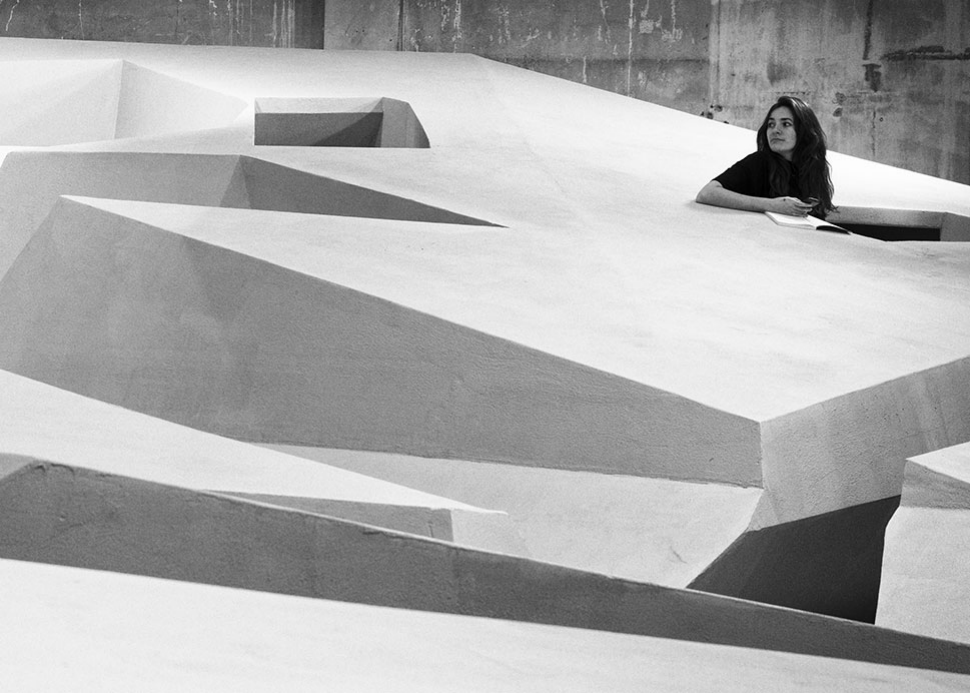
The End of Sitting–a closer view. Photograph reproduced with permission from Ricky Rijkenberg

This kind of empirical research is crucial for improving the landscape. In fact, this project integrates insights from several disciplines to bridge the gap between science and practice: visual art, architecture, empirical science (human movement sciences and ecological psychology) and philosophy (Figs. 8, 9). Within the field of philosophy, The End of Sitting is special in that it presents a philosophical worldview, however not in words, as philosophers typically do, but in the form of an enactive art installation. Rather than arguing for the claim that people are embodied minds situated in a landscape of affordances, this sculpture allows people to experience that physically in a landscape of *standing* affordances that gets them out of their comfort zone and confronts them with new possibilities for action to explore.

The End of Sitting is also a platform for scientific research on the office of the future. The subjects of the first empirical study by Withagen and Caljouw [14] reported that, compared with a traditional open office setting, The End of Sitting landscape was more pleasant to work in and better for their wellbeing [14]. For RAAAF and Barbara Visser these were important and encouraging findings. The architectural concept of *temporary comfort* of individual positions plus the variety of positions offered by the landscape, which makes switching possible, clarifies why Withagen and Caljouw [14] could observe that “many participants worked in several postures and changed location” in The End of Sitting. The installation manages to invite people to move more: only 17 % of participants worked in just one posture, which shows that most participants did indeed change, manifesting the dynamic of alternation of non-sitting postures we had aimed for. In addition, the subjects reported that their legs were more tired after working in the standing office, but that they felt more energetic after working in this new work landscape. Furthermore, the empirical study by Withagen and Caljouw [14] suggests that productivity in The End of Sitting was equal to that in the conventional office setting, but more research is needed to settle this matter. In summary, according to Withagen and Caljouw [14], The End of Sitting “naturally invit[es] changes in postures and thus movement” and “arguably promotes healthier behavior”. One of the most important open questions for future research on its health effects is what standing in this experimental working landscape means for metabolism of blood sugar and fat, as compared with sitting.

## Conclusion

Making people aware of the idea that relevant affordances drive our everyday behavior increases the chances that they start changing the material structure of the different places in which they spend their lives; replacing affordances that trigger unwanted, unhealthy or counterproductive activities with *new* ones. Replacing old affordances with new ones provides a way of thinking about scaffolding change in other domains of society as well. Discovering unorthodox affordances that can change our socio-cultural practices is *creativity* in action [21, 22]. Using this kind of discovery, we can make the transition from our sitting society to a more active and healthy society.
